# Analysis of Contact Deformations in Support Systems Using Roller Prisms

**DOI:** 10.3390/ma14102644

**Published:** 2021-05-18

**Authors:** Krzysztof Nozdrzykowski, Zenon Grządziel, Paweł Dunaj

**Affiliations:** 1 Faculty of Marine Engineering, Maritime University of Szczecin, 1-2 Wały Chrobrego St., 70-500 Szczeci, Poland; k.nozdrzykowski@am.szczecin.pl (K.N.); z.grzadziel@am.szczecin.pl (Z.G.); 2 Faculty of Mechanical Engineering and Mechatronics, West Pomeranian University of Technology, 19 Piastów Ave., 70-310 Szczecin, Poland

**Keywords:** crankshaft, support conditions, Hertz contact, asperity contact, V-block measurement, finite element analysis

## Abstract

This article presents the results of finite element analyses of the influence of reaction forces on stresses and strains at the contact points of the rollers of prism supports with cylindrical surfaces of the main journals of large-sized crankshafts. The analyses of strains and stresses, as well as the depth of their occurrences, in the case of the shaft journal and support rollers were carried out using Hertz contact theory and the finite element method. These calculation results proved to be highly consistent. Additionally, they provide a basis for stating that, in the case under consideration, permanent deformations do not significantly affect the values of the measured geometrical deviations nor the profile forms of the supported main crankshaft journals.

## 1. Introduction

A crankshaft is responsible for the correct operation of an engine and the conversion of linear motion into rotary motion [1]. Therefore, it should meet strict functional requirements, such as a very high fatigue strength [2,3] and wear resistance [4,5]. In addition to functional requirements, the final microstructure of a journal surface is very important, as it directly affects the service life of a crankshaft [6,7,8].

After a crankshaft has been manufactured, it should be properly verified to ensure that it meets the geometrical specification of the product. One of the commonly used methods of fixing and supporting cylindrical machine elements during measurements, is to support them in prisms [9]. This method is particularly attractive when measuring elements with large masses and dimensions [10]. The rationale for using prisms is their simple design, the ability to transfer significant loads, as well as the ability to rotate objects in them during measurements (especially in the case of roller prisms) [11,12].

In the case of fixing in prisms, unambiguous determination of the reference element is troublesome due to the shaft displacements that occur during its rotation [13]. These displacements depend on the dimensions of the shaft as well as the support method. This makes assessment of the geometry of the shaft in these conditions unreliable [14]. Therefore, predicting the impact of these phenomena on the measurement results is a key issue; this can be done based on an analysis of contact phenomena [15].

Zhang et al. [16] presented a use of X-ray computed tomography for the direct measurement of the contact phenomenon of rough surfaces. The actual contact area, along with a three-dimensional separation of the contact surfaces, which were made of aluminum and polycarbonate, were experimentally measured. The authors developed finite element models to determine the contact behaviors of the analyzed contact pairs. The obtained finite element results showed a good agreement with the experimental data.

Amor et al. [17] presented finite element analyses of elastic and elastic–plastic contacts between a rigid flat surface and a rough surface. The analyses considered the asperities interaction. The authors performed finite element modeling and measurements of the normal interfacial stiffness. Authors then investigated surfaces with different roughness values using the elastic and power-law hardening models to emphasize the combined effect of the topography and strain hardening on the contact characteristics. The obtained results showed the importance of considering strain hardening in the modeling of a rough contact, especially in case of a rougher surface.

Aidoudi and Bessrour [18] presented finite element modeling that considered the effect of surface roughness in elastic–plastic microcontacts between two cylinders that were in contact along their external generatrixes. The conducted analyses included modeling of the two types of frictionless contacts: (i) between two random rough cylindrical surfaces and (ii) between a total cylindrical surface, which included the roughness and the elasticity of both surfaces that were in contact, with a perfectly smooth rigid cylindrical surface. Results showed that the actual contact area ratio increases with the increase in displacement. This dependency is correct until it reaches a maximum limit value for elastic–plastic deformation.

Saha and Jackson [19] proposed a finite element model representing axisymmetric elastic–plastic sinusoidal surfaces in contact with a rigid flat surface. The presented model considered the effects of interactions with adjacent asperities. The obtained results showed that, for cases were amplitudes fall below the critical value, and are elastic in nature, that the previously published perfectly elastic model can be used.

An et al. [20] presented a novel microcontact stiffness model. The model involved the development of rough surfaces through the use of a cosine curve-shaped asperity and the Gauss distribution. Based on developed surface, an analytical model of the microcontact stiffness was formulated. Experimental verification showed that the model can accurately describe the contact stiffness between the grinding surfaces of steel materials.

Wen and Tang [21] presented a study of the contact between rough cylindrical surfaces considering elastic–plastic deformations of asperities. The authors considered the elastic deformation of a curved surface. The contact area was discretized with calculus. Then, on that basis, the nominal distance between the two surfaces was determined via iteration after assuming the pressure distribution. The analyses showed that, considering the elastic–plastic deformation of asperities in modeling, the contact between rough cylindrical surfaces was justified, as demonstrated by comparing the proposed model with others known in the literature.

Wang et al. [22] presented a numerical model for rough surface contacts. The developed model was applied to the wear prediction of spur gears. To obtain contact pressure, the authors discretized the integral equation, that the contact pressure should satisfy a set of linear equations. The proposed contact model was verified by comparing smooth and rough surface contacts. Moreover, the wear depths of a spur gear drive with geometry and pressure updates were analyzed. The results showed that a small roughness value between contact surfaces may cause a significant change in contact pressure.

A review of the literature shows that considering the roughness of contact surfaces is of great importance. Therefore, this approach was used in this article, and the influence of the contact between the shaft journal and the rollers of the measuring prisms on the results of crankshaft journal roundness measurements was examined. The article presents finite element analyses of the stresses and deformations of a rough shaft journal in contact with the smooth rollers of prisms made of steel and bronze. The obtained results were compared with analogous analyses for a smooth shaft journal. The influence of the shaft weight and contact deformations on the results of roundness measurements were examined using an outlined analysis.

This paper is structured as follows: Section 2 contains a description of the research problem and a finite element model is built. In Section 3, the results of the analyses and a proper discussion are provided. The main conclusions are presented in Section 4.

## 2. Materials and Methods

### 2.1. Research Problem

**Figure 1:**
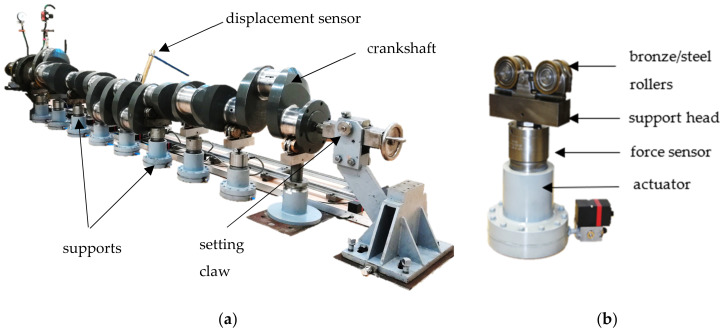
Measuring system (**a**) with elastic support of the crankshaft (**b**).

Depending on the adopted support conditions, during the measurement of shafts, deflections and elastic deformations occur. These issues are of particular importance in the case of measuring large-sized, slender shafts, such as the crankshafts of marine engines. Depending on the adopted support conditions, deflections and elastic deformations of the shaft can affect the results of measurements in terms of geometric quantities. This makes evaluation of the geometry of the shaft in these conditions unreliable. An effective method in eliminating deflections and elastic deformations of shafts is the use of a measurement system equipped with a so-called flexible support for the measured object. Such a system is presented in Figure 1.

One of the most important elements of the proposed system is the elastic support. The purpose of the elastic support is to implement variable values of reaction forces at the contact of rollers with the main journals during crankshaft rotation. The implemented values of the reaction forces guarantee zero deflection values on the individual supported main journals. The rollers are mounted on pneumatic cylinders and a force sensor is placed between the actuator and the rollers. The actuators are equipped with precise current-controlled valves, cooperating in a feedback system with force sensors, thanks to their implementation, variable reaction forces can be performed in a controlled manner.

The support heads are constructed in the form of roller prisms, as such, there are minimum values of frictional resistance at the contact points between the rollers with the journals, corresponding to the rolling friction resistance. The resistances depend on the value of the forces occurring at the contact between the rollers with the pin, and thus on the value of the force activated by the support. The problem of surface pressures and stresses is directly related to the problem of forces and frictional resistance at the roller–journal contact. By analyzing the impact of frictional resistance on the results of measurements of geometric quantities, it was found that the impact was negligible. However, a separate problem is the influence of surface pressures.

These pressures may cause, not only elastic deformation of the material of the pin or roller, but also a permanent deformation of cooperating elements. From the point of view of the measurement technique, including measurements of the deviations and shape contours, the influence of this factor on the measurement of geometrical quantities cannot be ignored and it should be individually analyzed, depending on the parameters of the measuring system and those of the measured object.

As emphasized earlier, a feature of the presented measurement system is that it allows to eliminate the elastic deformations of the crankshaft, thanks to the support system which realizes variable reaction forces that guarantee zero deflection values on the supported main journals. Therefore, it is justified to carry out an analysis of the influence of reaction forces on elastic or permanent deformations in the contact places of the rollers of the prism supports with the cylindrical surface of the supported main pins.

### 2.2. Reaction Forces Determination

Considering the above, the required values of the reaction forces were determined for the adopted test object—a crankshaft from a medium-speed main drive engine from a Buckau Wolf R8 DV136 ship, which is 3630 mm long and weighs 9280 N, with ten main journals with diameters of 149 mm and eight crank journals with a 144-mm diameter. The determination of the reaction forces was carried out using the finite element method.

**Figure 2:**
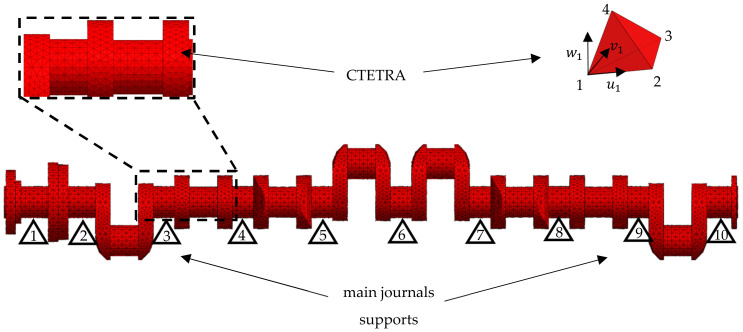
Finite element model of the analyzed crankshaft with the adopted numbering of the supported main journals.

Modeling was carried out using Midas 2019 (Midas Information Technology Co. Ltd., Seongnam, Korea) [23]. The finite element mesh used in the analyses was composed of tetrahedral elements (CTETRA), characterized with three translational degrees of freedom in each node. In summary, a finite element model with 137,475 finite elements and 126,114 degrees of freedom was obtained. The modeled crankshaft, with the adopted support numbering (corresponding to the fixed model constraints), is shown in Figure 2.

Next, a gravity load was applied to the model. An analysis consisting in the determination of shaft deflections and reaction forces acting on a supported shaft (force values were calculated when the shaft rotation angle was changed every 15°) was performed using the linear static Nastran solver (SOL101 [24,25]). The detailed modeling procedure can be found in [26].

**Figure 3:**
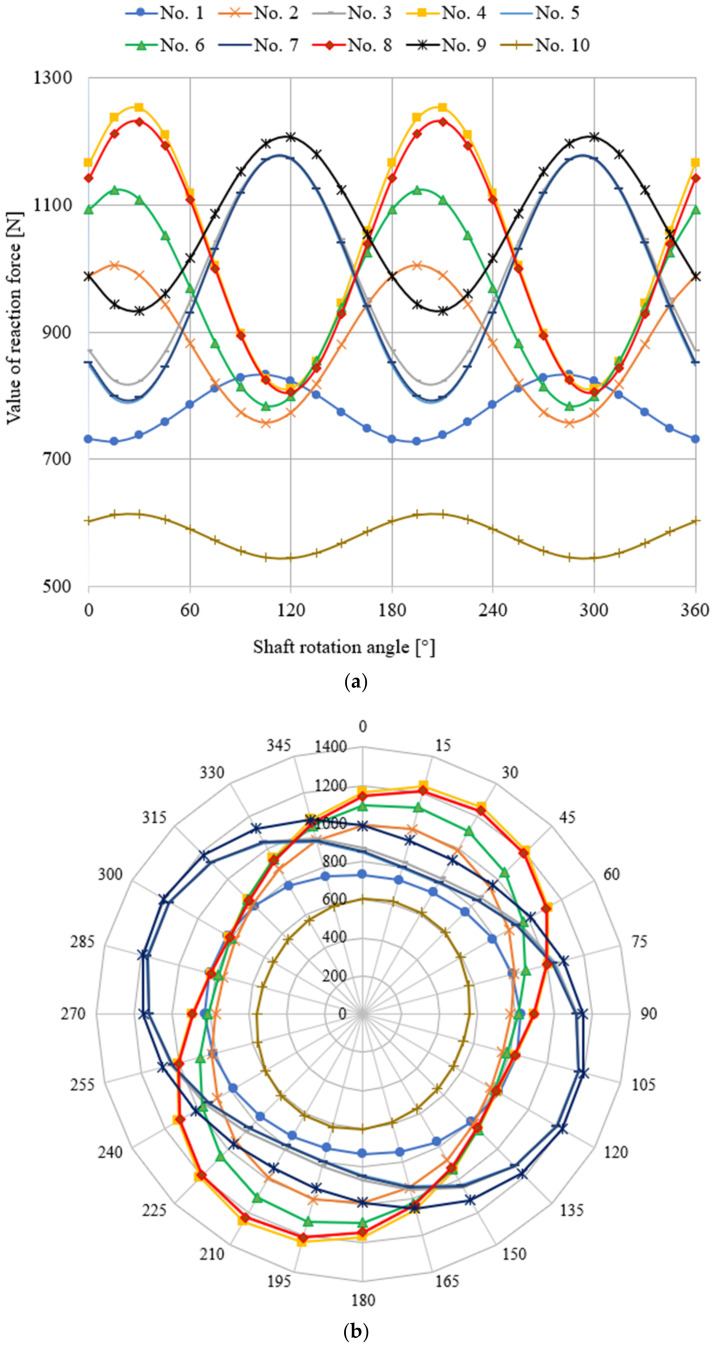
Distribution of the reaction forces guaranteeing zero value of deformations on the main pins of the crankshaft of a Buckau Wolf R8DV-136 motor. Diagrams of the coordinate systems: (**a**) Cartesian and (**b**) polar.

Based on the model, calculations were made for the reaction forces, guaranteeing the elimination of the deflections on the main journals; a graphical presentation of this is shown in Figure 3.

According to Figure 3, the maximum value of the reaction force occurred on journal No. 4 at shaft rotation angles of 30° and 210°, and amounted to 1253.23 N. Accordingly, the minimum value of the reaction force occurred on journal No. 10 at shaft rotation angles of 120° and 300°, and amounted to 545.54 N.

### 2.3. Finite Element Model for Contact Analysis

**Figure 4:**
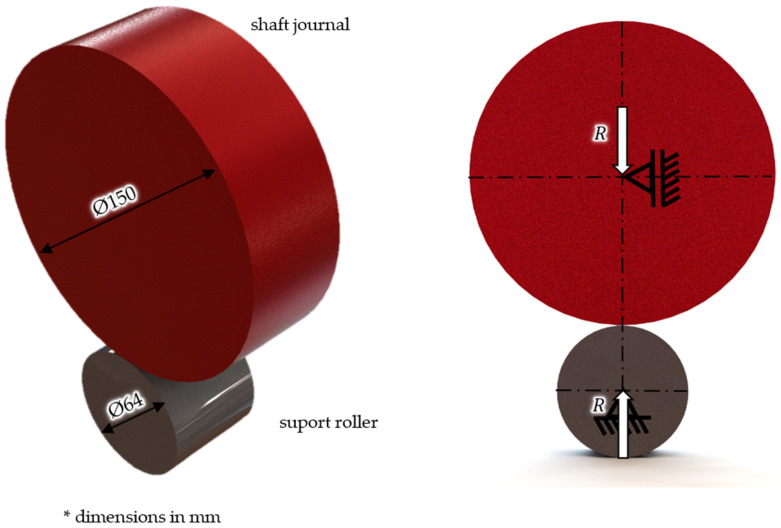
The cooperation model adopted for the analysis of surface pressures and stresses at the contact point of the rolling prism roller with the main shaft journal.

According to the diagram presented in Figure 4, the considered system corresponded to the cooperation of two rollers with the following parameters: roller diameter 64 mm and journal diameter 150 mm, which were in line with each other over 14 mm. It was also assumed that the values of the pressures and stresses were the result of the reaction forces which were equal to 1253 N, which acted on a single prism roller, and was classified in the upper limits of the calculated reaction forces for this crankshaft.

Next, the Hertz formulas [27] were used to determine the values of the surface pressures of cooperating elements. Accordingly, in the calculations it was assumed that, in the first of the considered cases of cooperation, both the shaft and the support roller were made of steel with a Young’s modulus *E* = 210 GPa and Poisson ratio *ν* = 0.28. In the second case, the shaft was made of steel with the above-mentioned material properties, while the support roller was made of aluminum–bronze with a Young’s modulus *E* = 210 GPa and Poisson ratio *ν* = 0.34.

The calculated stress values for the shaft journal were: in the case of a steel shaft cooperating with rollers of prisms made of steel, 215.2 MPa, while in the case of a steel shaft cooperating with rollers made of bronze, 175.3 MPa.

The presented results of the calculations of stresses and the depth of their occurrence are correct in terms of the linearity of stresses as a function of elongations (the applicability of Hooke’s law).

**Figure 5:**
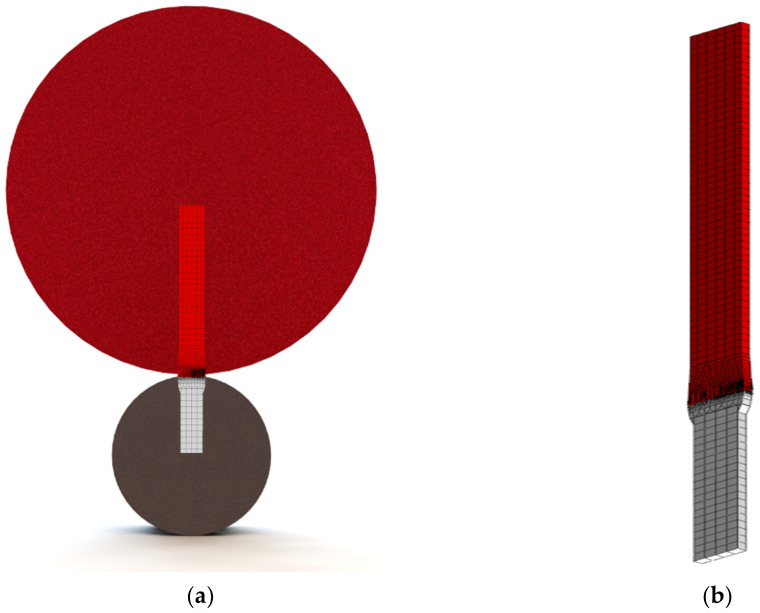
Scheme of the shaft–roller section against the background of both wheels, showing the shaft and the roller (**a**). The adopted model of cooperation between the shaft section and a 1-mm thick V-block (**b**).

To build a reliable finite element model for the analysis of contact phenomena, the model had to demonstrate the compliance of the obtained calculation results with the results obtained from theoretical formulas in the field of linearity. Obtaining this compliance was the basis for switching to calculations in the nonlinear range, where permanent deformations appear. The necessity to take finite element analysis of the contact between the surfaces of the shaft journal and the support roller into account required the modeling of elements to the centers of rotation of the shaft and the support roller (Figure 5).

**Figure 6:**
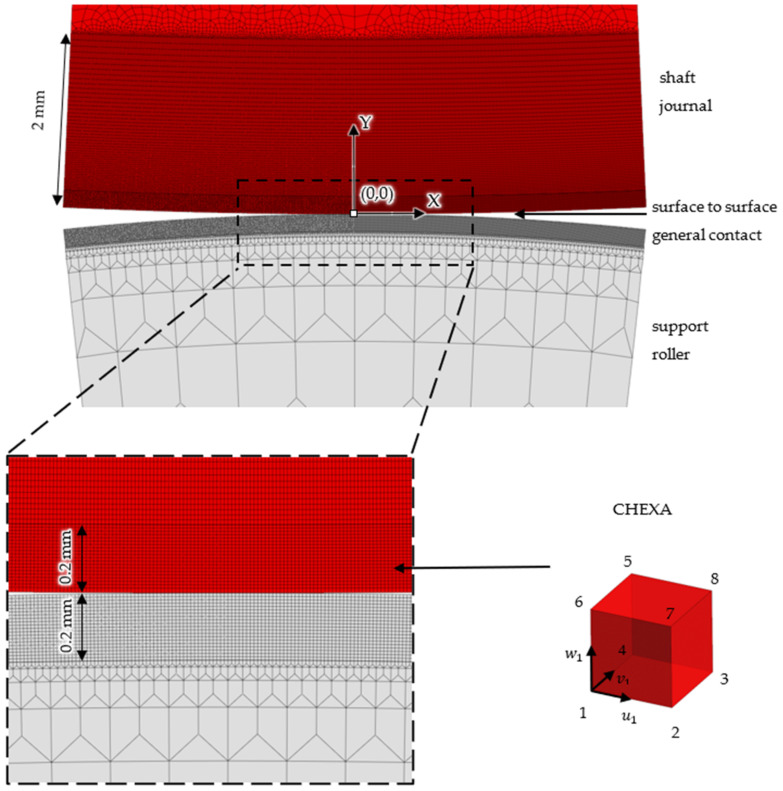
Division with finite elements with a side of 0.01 mm, near the contact of the shaft with the support roller.

The finite element model for contact analysis was build using eight-node six-sided isoparametric finite elements, and CPENTA six-node five-sided isoparametric elements. The model results consisted of 58,184 finite elements and 351,474 degrees of freedom. The shaft modeling part consisted of 44,312 finite elements and that of the roller consisted of 13,872 finite elements. In the contact area of the shaft and the support roller, a refined mesh was used to accurately reflect the values of the maximum contact stresses and the depths of their occurrences [28]. The results of the stress value calculations, carried out based on Hertz theory, showed that the occurrences of the maximum reduced stresses and the shear stresses were at depths of 100–150 μm. Hence, the side size of the finite element was set as 0.1 of the depth, i.e., 0.01 mm. The contact areas of the finite element model used for further analyses are shown in Figure 6. Since the surface of the shaft and its surface layer were treated as areas of permanent (plastic) deformation, a dense mesh division was maintained at a depth of about 2 mm. In the case of an supporting roller, where the formation of permanent deformations was not expected, it was not necessary to use a dense division at a depth greater than 0.2 mm.

## 3. Results and Discussion

Based on the built finite element model, the case of the shaft journal and support roller made from steel using the values of the maximum stresses and the depths of their occurrence with the reaction force *R* = 1253 N was determined (using non-linear Nastran solver—SOL106) [25] and verified using the Hertz contact theory. 

Considering the analyzed case from the point of view of the Hertz contact theory, it can be reduced to the contact analysis of two cylinders; according to which, Hertz contact stress *P*_0_ is determined as follows:

(1) $$P_{0}=\frac{4P_{mean}}{\pi },$$

where *P_mean_* is:

(2) $$P_{mean}=\frac{R}{2Bb},$$

contact area radius *b* is given by:

(3) $$b=2\sqrt{\frac{2Rr}{\pi BE^{\prime }}},$$

equivalent radius *r* is given by:

(4) $$r=\frac{r_{1}r_{2}}{r_{1}+r_{2}},$$

and reduced elastic modulus *E*′ is defined as follows:

(5) $$E^{\prime }=\frac{E_{1}E_{2}}{\left(1-\nu_{1}\right)E_{2}+\left(1-\nu_{2}\right)E_{1}}.$$

Based on this, it is possible to determine the values and locations of the maximum shear stresses and the maximum von Misses stresses. The detailed procedure for determining these values is presented in [29].

A comparison of the results of the finite element analyses and the Hertz contact theory is presented in Table 1.

**Figure 7:**
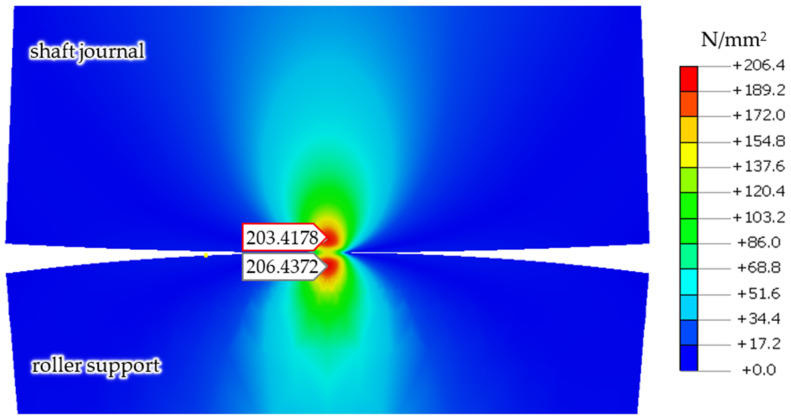
Calculation results of the finite element model, showing reduced stresses in the shaft and support roller, according to the von Mises hypothesis, for the case where a shaft and support roller are made of steel, with a reaction force *R* = 1253 N.

Figure 7 presents finite element calculation results, showing the values of reduced stresses in the shaft and support roller, according to the von Mises hypothesis, for the case where the shaft and support roller were made of steel, under the action of a reaction force of *R* = 1253 N.

Table 2 presents the analogous results for the steel shaft journal and the bronze support roller, with a reaction force of *R* = 1253 N.

**Figure 8:**
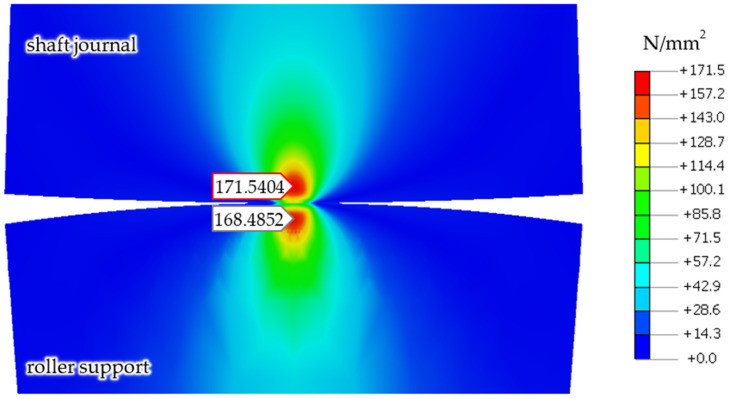
Calculations, showing reduced stresses in the shaft and support roller according to the von Mises hypothesis, for the case of a steel shaft and a support roller made of aluminum–bronze, under a reaction force of *R* = 1253 N.

Finite element calculation results, showing reduced stresses in the shaft and support roller according to the von Mises hypothesis for the case of a steel shaft journal and a bronze support roller, under the action of a reaction force of *R* = 1253 N are shown in Figure 8.

As stated earlier, for the steel of the shaft the Young’s modulus was *E* = 210 GPa, while the yield stress was *R_e_* = 250 GPa and the Poisson ratio was *ν* = 0.28. In terms of elasticity, the material tensile diagram is rectilinear with a steep inclination angle, the tangent value of which, in the adopted reference frame, corresponds to a Young’s modulus of *E* = 210 GPa. The steeply sloping waveform is valid until:

(6) $$\varepsilon \;=\frac{R_{e}}{E}=\frac{250}{210,000}=0.00119.$$

**Figure 9:**
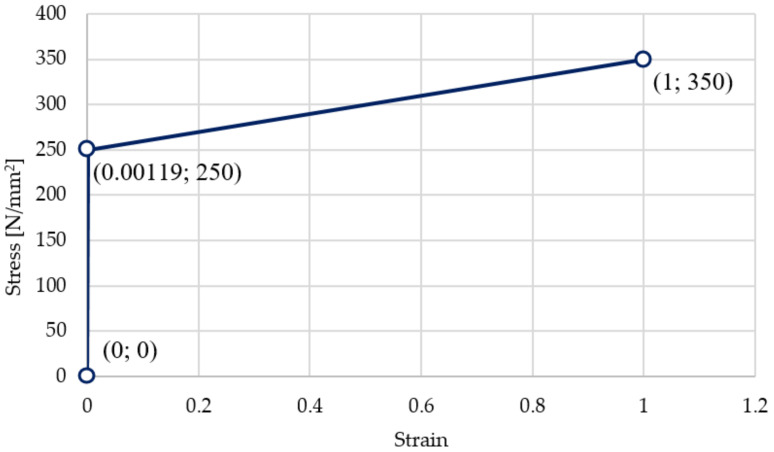
Tensile diagram in sigma–epsilon coordinates for the steel shaft.

Above the yield point, the tensile diagram is still rectilinear, but with a smaller inclination angle, the tangent of which, in the adopted reference frame, corresponds to a value of 100 MPa (Figure 9).

**Figure 10:**
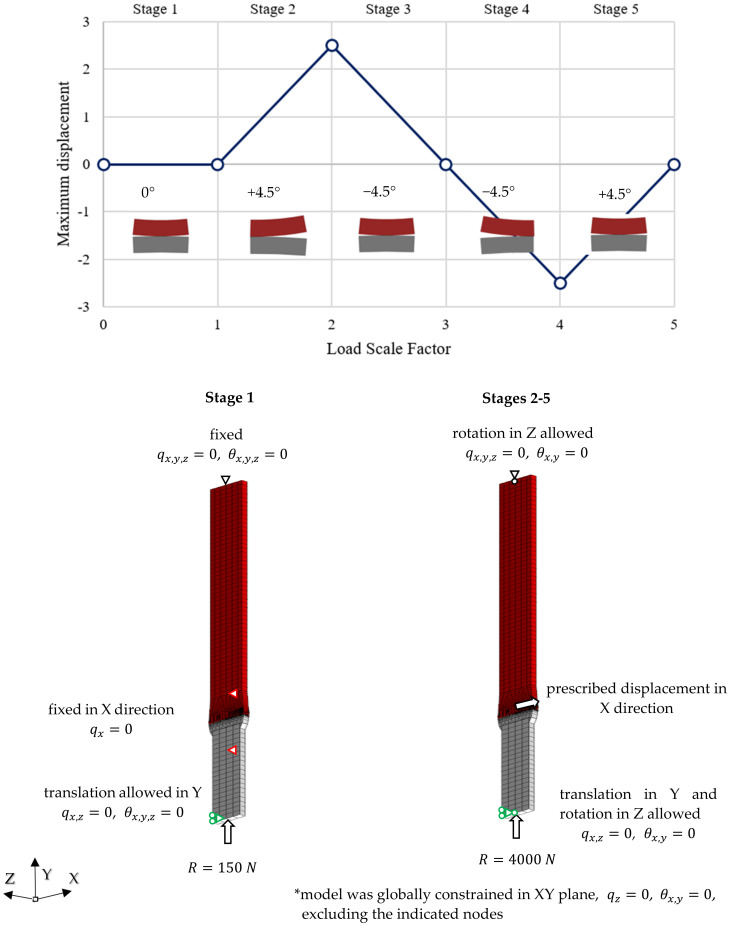
Stages of simulation calculations when shifting the shaft journal over a support roller over a distance of 5 mm.

Research in the field of non-linearity included a simulation of contact cooperation during rolling of the shaft journal over the support roller for a distance equal to 5 mm, which corresponded to an angular rotation of the roller equal to approximately 9°. Since the assumed initial value of the reaction force was equal to 1253 N, there were no permanent deformations and reaction force R was increased to 4000 N (simulating a larger or more slender shaft). The calculations were carried out in five successive stages (Figure 10):

- Stage 1—initial shaft pressure on roller the with a force of *R* = 150 N, “zero” position;
- Stage 2—rotation of the shaft by an angle of +4.5°, a gradual increase in the pressure force to a value of *R* = 4000 N;
- Stage 3—rotation of the shaft by an angle of −4.5° under the pressure of the force *R* = 4000 N, “zero” position;
- Stage 4—rotation of the shaft by a further angle of −4.5° under the pressure of the force *R* = 4000 N;
- Stage 5—rotation of the shaft by an angle of +4.5° and a gradual decrease in the pressure force to *R* = 0 N.

With these stages, the cycle of turning the shaft journal “back and forth” over the supporting roller over a distance of 5 mm was carried out. The calculations with the use of the non-linear Nastran solver—SOL106 required 5 iterations in stage 1 and the 50 iterations in the remaining stages. The change in the angular position of the shaft was simulated using a prescribed displacement, which, according to the adopted boundary conditions, resulted in shaft mesh rotation.

The “turning” procedures were carried out for a shaft journal with an ideal circle profile (not burdened by irregularities in the shape outline), and a shaft journal with a real profile, with an irregular roundness outline. These procedures were carried out, as previously mentioned, for the cooperation of the shaft journal and the steel supporting roller, and the steel shaft journal and the supporting roller made of aluminum–bronze.

**Figure 11:**
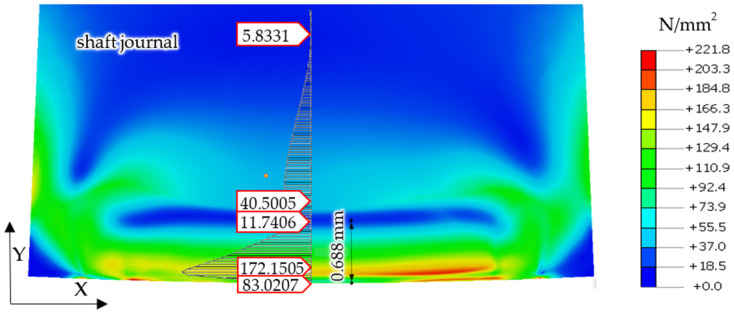
Distribution of permanent stress values in the surface layer of the steel shaft journal, not burdened with outline irregularities, after the outline has been turned over the steel supporting roller.

**Figure 12:**
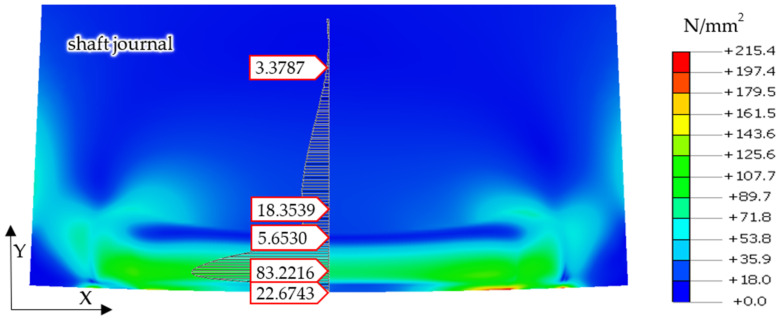
Distribution of permanent stress values in the surface layer of a steel shaft journal with a profile free of outline irregularities after the outline has been turned over a support roller made of aluminum–bronze.

Therefore, considering that there are no permanent deformations in the support roller, the stress analyses were limited to residual stresses occurring in the surface layer of the shaft. Of course, not only were the stress values analyzed, but the permanent deformations were analyzed as well. Figure 11 and Figure 12 show graphical presentations of the obtained research results.

After turning the steel journal with no irregularities over the steel support roller with a force of *R* = 4000 N, permanent stresses remained in the surface layer of the shaft material. The distribution of these stresses is shown in Figure 11.

A similar form of permanent stress was shown in the results of the analyses for the case of the cooperation of the steel shaft journal, which is not burdened with irregularities in the profile, with the bronze support roller with a force of *R* = 4000 N. Permanent stresses remain in the surface layer of the shaft material, the distributions of which are shown in Figure 12. However, the values of these stresses are much smaller and their reduction in relation to the obtained stress values for the case of a steel support roller cooperating with a steel shaft journal exceeded 50% (Figure 12).

**Figure 13:**
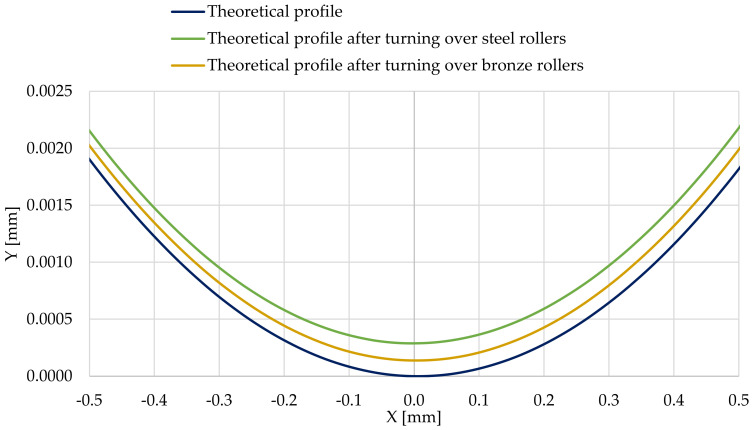
Comparison of the profile of the steel shaft journal, which is not burdened with outline irregularities, with the profiles after the outline has turned over the steel support roller and the support roller made of aluminum–bronze.

Figure 13 shows a comparison of the steel shaft journal profile, which is not burdened with outline irregularities, after the outline has been turned over the steel support roller and the support roller made of aluminum–bronze.

**Figure 14:**
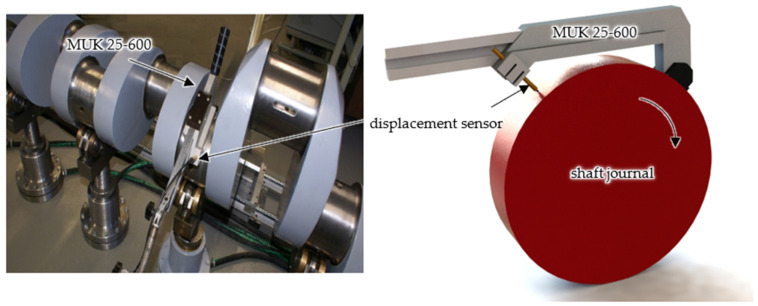
SAJD measurement system.

In the case of a shaft journal with an irregular outline, the actual profile of journal No. 10 was selected for the analyses of permanent stresses and deformations. The actual measured profile of journal No. 10 was recorded during 1024 measurements with equidistant points located on its circumference. The detailed procedure and description of the SAJD measurement system can be found in [30]. The SAJD measurement system is shown in Figure 14.

**Figure 15:**
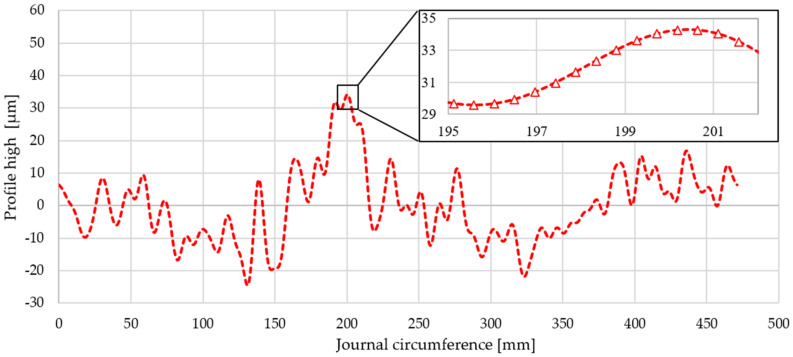
Actual measured outline of the roundness of journal No. 10 developed over a length equal to 471 mm, which corresponds to the circumference of a wheel with a nominal diameter of the measured main shaft journal of 150 mm.

This profile was evenly distributed over a length of 471 mm, which corresponded to the circumference of a wheel with the nominal diameter of 150 mm for the measured main journal (Figure 15).

**Figure 16:**
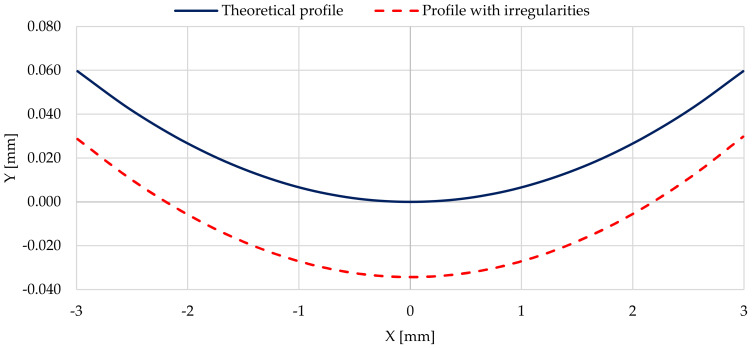
Profiles: not burdened with irregularities and actual one around coordinate *x* = 200 mm (from Figure 15), transformed into coordinate *x* = 0.

In the tests of permanent stresses and deformations of the actual profile, they were limited to a segment of the actual shaft profile, near the contact with the support roller around coordinate *x* = 200 mm. This coordinate determined the maximum height of the measured profile, equal to 0.035 µm. An enlarged fragment of this selected section of the real profile, 6 mm long (from −3 mm to 3 mm), was transformed into the new coordinate of the peak occurrence, *x* = 0 mm, and was presented against the background of the shaft profile with no outline irregularities (shown in Figure 16).

**Figure 17:**
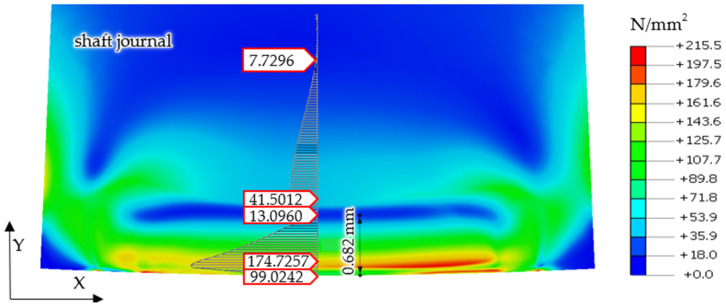
Distribution of permanent stress values in the surface layer of the steel shaft journal, with the actual profile of journal No. 10, after this profile has been turned over the steel support roller.

Figure 17 shows the distribution of permanent stress values in the surface layer of the steel shaft journal, with the actual profile of shaft journal No. 10, after the profile has been turned over the steel supporting roller.

**Figure 18:**
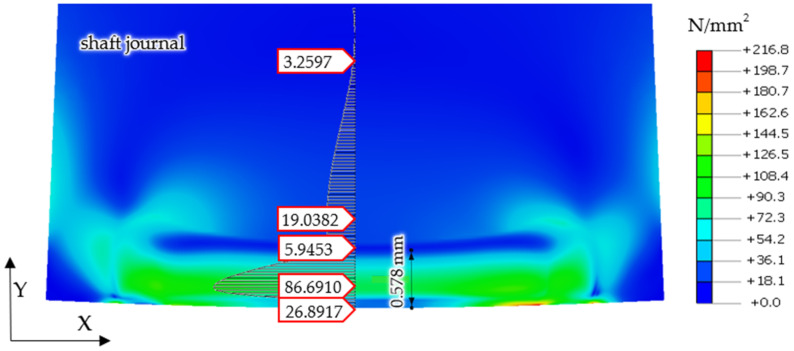
Distribution of permanent stress values in the surface layer of the steel shaft journal with actual journal profile No. 10, after this profile has been turned over the support roller made of aluminum–bronze.

The distribution of the permanent stress values in the surface layer of the steel shaft journal with the actual profile of journal No. 10, after this profile has been turned over the support roller made of aluminum–bronze (shown in Figure 18).

**Figure 19:**
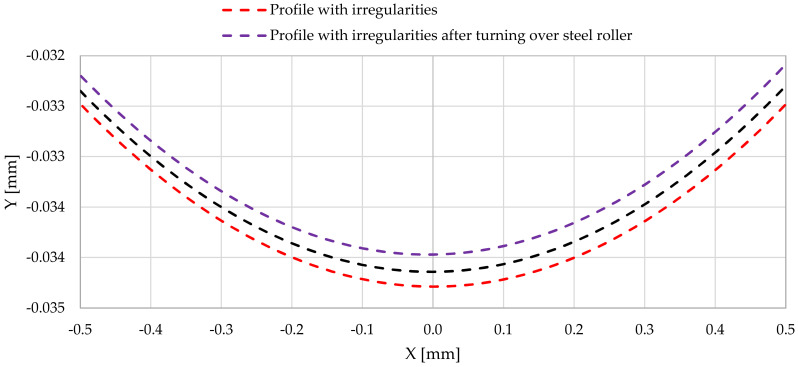
Comparison of the actual profile of a steel shaft with the profiles after the actual outline has been turned over a support roller made of steel and a support roller made of aluminum–bronze.

Figure 19 presents a comparison of the actual profile of the steel shaft with profiles after the actual outline has been turned over the steel support roller and the support roller made of aluminum–bronze.

**Figure 20:**
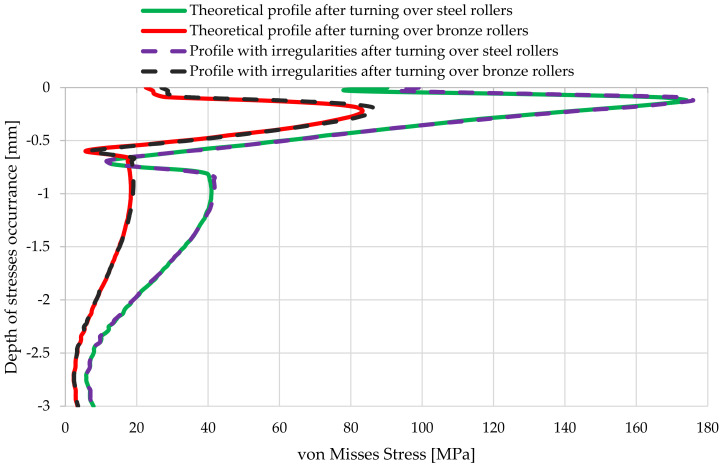
Graphs of von Mises stresses remaining in the steel shaft journal as a function of the depth of their occurrence, for the “zero” position at the reaction force of *R* = 4000 N for the journal profile not burdened with outline irregularities and the actual one, after turning these outlines on a steel support roller and a support roller made of aluminum–bronze.

A cumulative comparative diagram of the reduced stresses according to von Misses, remaining in the shaft journal made of steel as a function of the depth of their occurrence is shown in Figure 20; the stresses for the “zero” position at reaction force *R* = 4000 N, for the journal profile not burdened with outline irregularities, and the actual one after turning these contours on support rollers made of steel and of aluminum–bronze are shown.

## 4. Conclusions

This article presents the results of analyses on the influence of reaction forces on the stresses and strains at the contact points of the rollers of prism supports with cylindrical surfaces of the main journals of a large-sized crankshaft.

The finite element models used in the research were verified using the Hertz contact theory in terms of the values and locations of the maximum stresses, and showed a high consistency. Comparing the accuracy of the developed models to those presented in the literature, and also verified based on the Hertz contact theory, it can be deduced that similar results were achieved [31,32,33].

Then, based on the verified finite element model, nonlinear static analyses was carried out, including the rolling of smooth and irregular shaft journals over steel and bronze rollers. The results of the analyses showed that rolling a shaft with irregularities on the steel support roller caused large permanent deformations on the surface of the shaft journal that were almost twice as compared to the case of rolling on a roller made of aluminum–bronze. This also applied to turning of the shaft journal, not burdened with outline irregularities (Figure 13 and Figure 19). The maximum peak of the actual profile from Figure 15 and Figure 16 turns out to be very mild due to the formation of additional reduced stresses (Figure 20). After the steel shaft journal with a profile free of outline irregularities was turned over the support roller made of steel, the maximum reduced permanent stress at the central point was 83 MPa (Figure 11), and after the actual profile turns, the stress increased to 99 MPa (Figure 17). After the steel shaft journal with a profile free of outline irregularities was turned over the support roller made of aluminum–bronze, the maximum reduced permanent stress at the central point was 22.7 MPa (Figure 12), and after the steel journal with an actual profile was turned over in the support roller made of the same material, the stress increase to 26.9 MPa (Figure 18). These results qualitatively correspond to the results of similar studies [34,35].

The presented comparative assessment showed that the material used to make the roller has a large impact on the value of the permanent stresses; this is very visible in Figure 20. Regardless of the journal profile, almost the entire depth of the occurrence of stresses is reduced almost twice as much in the case of the steel support roller as compared to the support roller made of aluminum–bronze.

From the point of view of the aim of the undertaken research, there is no concern that, during the measurements of the tested objects, permanent deformations may occur because of the movement cooperation of the rollers of the prism supports with the supported crankshaft journals. Thus, it can be assumed that, in the case under consideration, permanent deformations do not significantly affect the values of the measured geometrical deviations and the outlines of the shapes of the main journals of the crankshaft. Therefore, it can be concluded that the aim of the study has been achieved.

Further research, based on the model presented in this article, will include comprehensive modeling of the system presented in Figure 1 and its experimental verification.

## Figures and Tables

**Table 1:** Summary of the results of calculations of the maximum stresses and the depth of their occurrence for the shaft journal (SJ) and the steel roller support (RS), under a reaction force of *R* = 1253 N.

Quantity	Unit	Hertz Contact Theory	Finite Element Model	Relative Error %
Hertz contact stress	MPa	3.4	359.9	5.4
Max. shear stress RS	MPa	114.2	110.7	3.1
Max. shear stress SJ	MPa	114.2	108.6	4.8
Max. von Mises stress body RS	MPa	215.2	206.4	4.6
Max. von Mises stress body SJ	MPa	215.2	203.4	5.4
Max. shear stress location RS	μm	−117.8	−120.0	1.8
Max. shear stress location SJ	μm	−117.8	−120.0	1.8
Max von Mises stress location RS	μm	−102.8	−110.0	6.5
Max von Mises stress location SJ	μm	−102.8	−110.0	6.5

**Table 2:** Summary of the calculation results of the maximum stresses and the depths of their occurrence for the steel shaft journal (SJ) and the aluminum–bronze roller support (BRS), after a reaction force of *R* = 1253 N.

Quantity	Unit	Hertz Contact Theory	Finite Element Model	Relative Error %
Hertz contact stress	MPa	309.8	300.6	3.0
Max. shear stress BRS	MPa	93.03	93.7	0.7
Max. shear stress SJ	MPa	93.03	91.9	1.2
Max. von Mises stress body RS	MPa	168.5	168.5	0.0
Max. von Mises stress body SJ	MPa	175.3	171.5	2.2
Max. shear stress location BRS	μm	−144.6	−150.0	3.6
Max. shear stress location SJ	μm	−144.6	−152.0	4.8
Max von Mises stress location BRS	μm	−135.1	−130.0	3.8
Max von Mises stress location SJ	μm	−126.2	−128.0	1.4

## Data Availability

Data available on request due to restrictions, e.g., privacy or ethical.
